# Influence of volatile anesthesia versus total intravenous anesthesia on chronic postsurgical pain after cardiac surgery using the Initiative on Methods, Measurement, and Pain Assessment in Clinical Trials criteria: study protocol for a prospective randomized controlled trial

**DOI:** 10.1186/s13063-019-3742-4

**Published:** 2019-11-27

**Authors:** Hong Yu, Jian-Qiao Zheng, Yu-Si Hua, Shuo-Fang Ren, Hai Yu

**Affiliations:** 1 Department of Anesthesiology, West China Hospital, Sichuan University & The Research Units of West China (2018RU012), Chinese Academy of Medical Sciences, Chengdu, 610041 People’s Republic of China; 2 Department of Cardiovascular surgery, West China Hospital, Sichuan University & The Research Units of West China (2018RU012), Chinese Academy of Medical Sciences, Chengdu, 610041 People’s Republic of China

**Keywords:** Randomized controlled trial, Volatile anesthesia, Propofol, Total intravenous anesthesia (TIVA), Chronic postsurgical pain (CPSP), Initiative on methods, Measurement and Pain Assessment in Clinical Trials (IMMPACT) criteria assessment

## Abstract

**Background:**

Many patients develop chronic postsurgical pain (CPSP) after cardiac surgery, which interferes with their sleep, mood, and quality of life. Studies have suggested that propofol improves postoperative analgesia compared with volatile anesthetics, but its preventive effect on CPSP following cardiac surgery is still unknown. This study compares the incidence of CPSP following cardiac surgery for those receiving volatile anesthesia and those receiving propofol-based total intravenous anesthesia (TIVA) using criteria recommended by the Initiative on Methods, Measurement, and Pain Assessment in Clinical Trials (IMMPACT).

**Methods/design:**

This is a prospective randomized controlled trial. In total, 500 adults undergoing cardiac surgery will be randomly allocated to the volatile or the TIVA group. The volatile group will receive sevoflurane or desflurane during surgery as general anesthesia. The TIVA group will receive propofol-based intravenous agents and no volatile agents during surgery. The primary outcomes will be the frequency of CPSP at 3 months, 6 months, and 1 year after surgery. In this case, CPSP is sternal or thoracic pain. It is defined as either (1) numerical rating scale (NRS) > 0 or (2) meeting all six IMMPACT criteria for CPSP. The IMMPACT criteria are validated pain instruments.

**Discussion:**

To our knowledge, this is the first prospective randomized controlled trial to investigate the prevention of CPSP following cardiac surgery for patients receiving volatile anesthesia compared to those receiving propofol-based TIVA using validated pain instruments in accordance with the IMMPACT recommendations. This study will provide important information on which of these two anesthetic regimens is better for preventing CPSP after cardiac surgery.

**Trial registration:**

Chictr.org.cn, ChiCTR1900020747. Registered on 16 January 2019.

## Background

Cardiac surgery is one of the most common forms of major surgery, with over 2 million patients undergoing this procedure worldwide each year [1]. Many patients develop chronic postsurgical pain (CPSP), which can occur in the anterior thorax after a median sternotomy [1]. It is estimated that CPSP has an incidence of 11–56% in patients undergoing cardiac or thoracic surgery, depending on the study population and length of follow-up [2–5]. CPSP that persists after cardiac surgery is a major clinical problem, because it disturbs daily life and interferes with sleep, mood, and quality of life [6–8]. Considering the large number of patients who undergo cardiac surgery, identifying potential treatments for CPSP is important [9]. In addition to standard postoperative analgesics, it has been suggested that corticosteroids, N-methyl-D-aspartate (NMDA) antagonists, alpha-2 agonists, local anesthetics, and gabapentinoids can reduce the risk of CPSP after cardiac surgery [5]. However, no specific therapy has been demonstrated to protect against CPSP [10–12].

Patients undergoing cardiac surgery need general anesthesia from either intravenous (IV) anesthetics (such as propofol) or volatile anesthetics (such as isoflurane, sevoflurane, and desflurane). Studies have found that propofol has anti-inflammatory and antioxidative effects on the biosynthesis of cytokines, which are important in pain signaling [13]. Propofol’s ability to scavenge free radicals is useful and important. It has antioxidant properties and may also dynamically protect the body [14]. Moreover, propofol can modulate NMDA receptors in neurons in vivo, which play a crucial role in the transmission and maintenance of the pain signaling pathway [14, 15]. These anti-inflammatory, free radical scavenging, and NMDA receptor antagonistic properties of propofol imply that it may have a possible perioperative analgesic effect. The meta-analyses by Qiu et al. and Peng et al. suggest that propofol improved postoperative analgesia compared with inhalational anesthesia [16, 17]. However, most previous studies were not designed to detect differences in chronic pain [16, 17], and a few clinical trials investigating CPSP focused on non-cardiac surgery [18, 19]. To date, no published clinical trial has compared the effects of volatile anesthesia with those of propofol-based total intravenous anesthesia (TIVA) in preventing CPSP after cardiac surgery through sternotomy.

Prior studies focusing on CPSP after either cardiac or non-cardiac surgery assessed pain using a yes or no scoring system or a numerical rating scale (NRS) to evaluate the level of pain. Few studies have used the Initiative on Methods, Measurement, and Pain Assessment in Clinical Trials (IMMPACT) recommendations to evaluate CPSP [20]. The IMMPACT approach to assessing CPSP evaluates the quality of the pain, the degree of pain at rest and during movement, the clinical meaningfulness of the pain, and the influence of pain on physical and emotional functioning, instead of focusing on only the absence or presence of pain [20–23]. Thus, the aim of this randomized controlled trial is to assess the prevention of CPSP following cardiac surgery through sternotomy for patients receiving volatile anesthesia compared to those receiving propofol-based TIVA using validated pain instruments in accordance with the IMMPACT recommendations.

## Methods/design

### Study design, approval, and registration

**Fig. 1:**
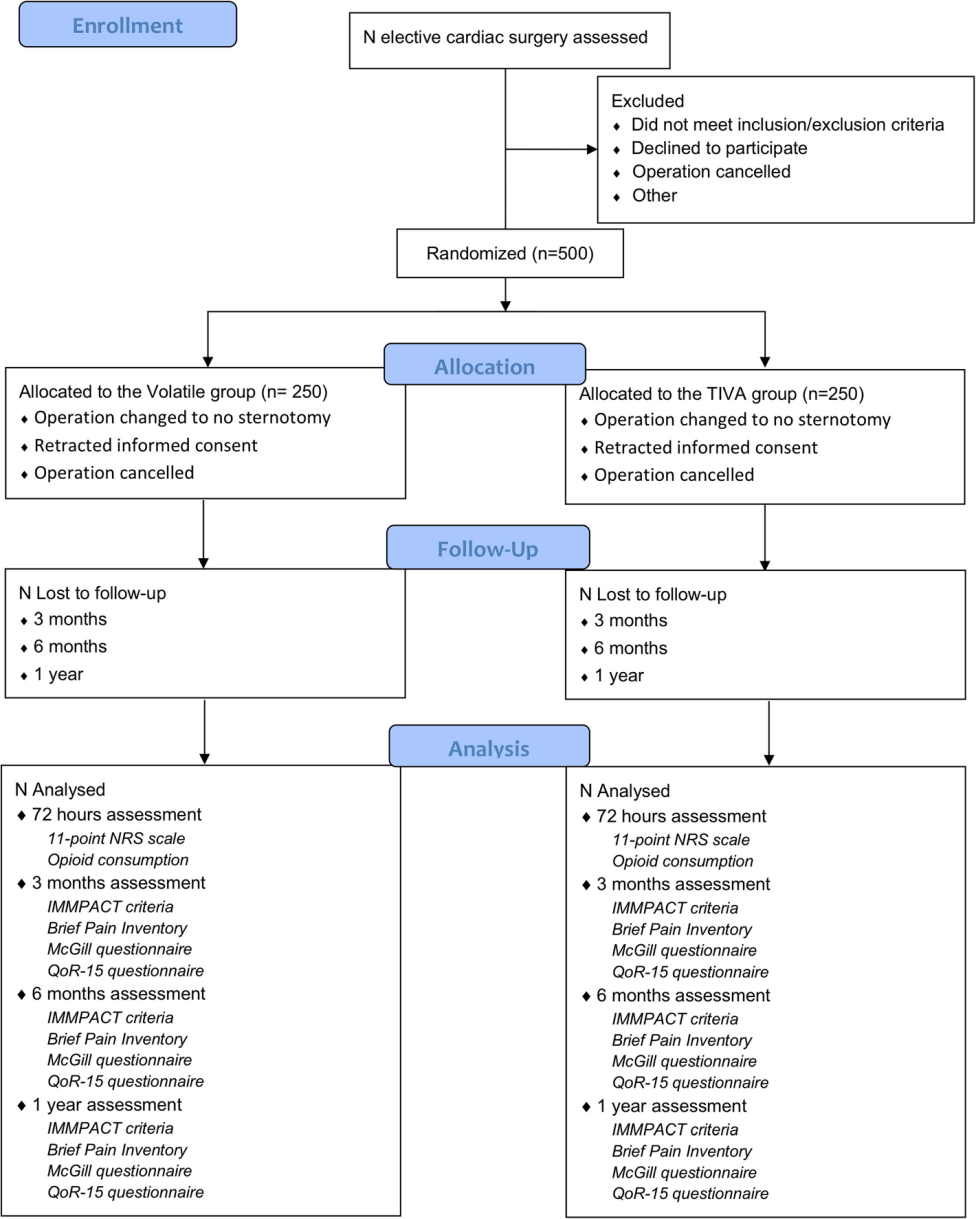
Consort diagram of study participant flow. TIVA total intravenous anesthesia, NRS numeric rating scale, IMMPACT Initiative on Methods, Measurement, and Pain Assessment in Clinical Trials, QoR-15 15-item Quality of Recovery Questionnaire

The planned study is a parallel-group randomized controlled trial with a 1:1 allocation ratio undertaken in West China Hospital of Sichuan University. Figure 1 is the trial flowchart. Recruitment commenced in February 2019. Additional file 1 is the Standard Protocol Items: Recommendations for Interventional Trials (SPIRIT) checklist. The schedule of enrollment, interventions, and assessments follows the SPIRIT Figure (Additional file 2). The study has been approved by the ethics committee of West China Hospital of Sichuan University and has been prospectively registered at Chictr.org.cn (ChiCTR1900020747).

### Study aim

The aim of our study is to compare the incidence of CPSP following cardiac surgery for patients receiving volatile anesthesia and those receiving propofol-based TIVA using the IMMPACT criteria.

### Participants

We plan to enroll 500 participants aged more than 18 years undergoing a cardiopulmonary bypass (CPB) for any elective cardiac surgical procedure via a median sternotomy such as procedures that involve the valves, coronary arteries, or aorta, or combined procedures.

#### Inclusion criteria

Participants must meet all the inclusion criteria:

1. Aged older than 18 years
2. Undergoing a CPB for cardiac surgery via a median sternotomy
3. Signed the informed consent form

#### Exclusion criteria

Patients who meet any of the following criteria will be excluded from participation:

1. Those undergoing combined cardiac and non-cardiac surgery
2. Those undergoing emergency surgery
3. Pregnant women
4. Those with a suspected family history of malignant hyperthermia or propofol infusion syndrome
5. Those who fail to cooperate with the follow-up procedures due to a lack of understanding of the study procedures

### Randomization, allocation, and concealment

Once informed consent has been received and the preoperative assessments completed, patients will be entered into the trial. Subjects will be allocated using a web-based centralized dynamic randomization service. The dynamic randomization considers patient characteristics including age, gender, European System for Cardiac Operative Risk Evaluation (EuroSCORE) score, predicted CPB duration, and body mass index. The anesthesiologists will be aware of patients’ group allocation because they will provide the trial intervention, but they will not be involved in either the postoperative treatment or the analysis. Patients, intensive care physicians, data collectors, and outcome adjudicators are blinded to treatment allocation.

### Interventions

Patients who meet the enrollment criteria will be randomized 1:1 to either the volatile or the TIVA group. Three investigators (Hong Yu, JQZ, and YSH) will explain the treatment intervention in detail and supervise the compliance of the intervention throughout the entire procedure (from maintenance of anesthesia to transport to an intensive care unit).

#### The volatile group

The volatile group will receive sevoflurane or desflurane to provide general anesthesia during surgery from maintenance of anesthesia to transport to an intensive care unit, including during CPB. Maintenance of anesthesia in the treatment group uses sevoflurane or desflurane at a minimum end-tidal concentration of 0.5–2 minimal alveolar concentration throughout the entire procedure. During CPB, patients will receive sevoflurane or desflurane from a vaporizer connected to an air blender, which is connected to an oxygenator. The minimal alveolar concentration is measured at the outlet of the oxygenator of the extracorporeal circulation.

#### The TIVA group

The TIVA group will receive propofol at an infusion rate of 3–8 mg kg^− 1^ h^− 1^ with or without other IV agents. The only absolute criterion for this group is that no volatile anesthetic is to be used at any time during the procedure.

### Perioperative management

#### Induction of anesthesia

General anesthesia will be induced with midazolam, sufentanil, and propofol as necessary. Tracheal intubation will be facilitated with either rocuronium or cisatracurium. There is no restriction on the type or dose of anesthetic used to induce anesthesia.

#### Ventilation

Patients will be ventilated using a lung-protecting ventilation strategy before and after CPB. The pressure-controlled ventilation will maintain a tidal volume of 6–8 ml kg^− 1^ based on ideal body weight, a positive end expiratory pressure of 5–8 cm H_2_O, an inspiratory to expiratory ratio of 1:2; an inspired oxygen fraction of 0.4 to 0.8, and a respiratory rate of 10–16 per min adjusted to keep a desired end-tidal CO_2_ of 35–45 mmHg. A recruitment maneuver with a peak airway pressure of 30 cmH_2_O for 30 s, as an essential part of the protective ventilation strategy, will be performed before beginning and before discontinuing CPB and exiting from the operating room. The use of ventilation during CPB will be chosen by the anesthesia care providers.

#### Anesthesia maintenance

Propofol or inhalation anesthetics plus sufentanil and a nondepolarizing muscle relaxant will be used to maintain general anesthesia with dosages at the discretion of the attending clinicians. Sufentanil will be administered to avoid changes of mean arterial pressure of more than 20% from baseline while maintaining a mean arterial pressure of at least 65 mmHg. A vasopressor will be administered as necessary. Remifentanil will be administered at an infusion rate of 0.1–0.2 μg kg^− 1^ min^− 1^. The dosage of dexmedetomidine will be limited to less than 0.5 μg kg^− 1^ h^− 1^ if needed. No antiemetics will be administrated for nausea or vomiting prophylaxis.

#### Postoperative analgesia

After surgery, patients will be transferred to an intensive care unit for further care. Patients will receive an infusion of 10–25 μg kg^− 1^ h^− 1^ morphine or 100 mg of IV meperidine to maintain an NRS of less than 4 (0 = no pain, 10 = worst pain imaginable), as assessed by the nursing personnel. IV analgesia will be discontinued, and patients will be given oral celecoxib or ibuprofen when they can tolerate oral medication. Patient-controlled IV analgesia pumps will not be used.

### Data collection

#### Baseline characteristics of patients

Demographic data, cardiac history, coexisting medical conditions, comorbidities, smoking status, EuroSCORE score, depression and anxiety history, chronic pain at presentation in an area other than the operative site, surgical procedure, intraoperative sufentanil and remifentanil dosage, and health-related quality of life measured with the 15-item Quality of Recovery Questionnaire (QoR-15) [24] will be recorded.

#### Acute pain assessment at 24, 48, and 72 h after surgery

Patients will be visited and evaluated over the first 72 h after surgery. Pain is assessed using an 11-point NRS scale (0 = no pain, 10 = worst pain imaginable) at 24, 48, and 72 h after surgery. The amount of opioid analgesics consumed is determined from their electronic medical record and is converted to an equivalent dose of IV morphine [25].

#### Follow-ups at 3 months, 6 months, and 1 year

Each patient will receive follow-up phone calls at 3 months, 6 months, and 1 year after surgery to answer questions regarding the presence, quality, and severity of pain using the Brief Pain Inventory [26], the McGill Short Form Questionnaire [27], and the QoR-15 questionnaire [24]. Each patient will leave at least three phone numbers and receive a maximum of three telephone calls if contact could not be made.

### Outcomes

#### Primary outcome

The primary outcome is the occurrence of CPSP at 3 months, 6 months, and 1 year after surgery. CPSP is defined as sternal or thoracic pain that the patient identifies as related to their surgery in two ways: (1) NRS > 0 and (2) pain that meets all six IMMPACT criteria for CPSP. The IMMPACT criteria are validated instruments for assessing pain [28].

#### IMMPACT Questionnaires

Chronic pain is assessed in accordance with the IMMPACT recommendations in the following six domains [28]: (1) absence or presence of pain in the area of surgery, (2) clinically important daily average pain (NRS ≥ 4 on a 0- to 10-point scale), (3) clinically important pain at rest (NRS ≥ 4), (4) clinically important pain intensity upon movement or activity (NRS ≥ 4), (5) quality of pain, and (6) physical and emotional functioning [21, 22, 29]. The Brief Pain Inventory is used to determine the domains 1 to 4 and 6. It measures the intensity of daily average pain, pain at rest, pain during movement or activity, as well as physical and emotional functioning [26]. The McGill Pain Questionnaire is used to assess domain 5, which relates to the impact of both sensory and affective pain [27]. A total pain index score ≥12 is associated with chronic pain [30]. Subjects have to meet the threshold for all six outcome domains to fulfill the IMMPACT criteria.

#### Secondary outcomes

The secondary outcomes are: (1) NRS pain scores (0–10) 24, 48, and 72 h after surgery, (2) opioid consumption during the first 72 h after surgery, (3) the Brief Pain Inventory, the McGill Pain Questionnaire, and health-related quality of life measured with QoR-15 at 3 months, 6 months, and 1 year after surgery.

### Statistics

#### Sample size estimate

The sample selected for this study was based on the finding of our prior study exploring the incidence and possible risk factors of CPSP in patients undergoing cardiac surgery with CPB via a median sternotomy. That study showed that 60.9% patients had CPSP at 3 months postoperatively [31]. The sample size for the current study is 250 patients per group, for a total of 500 patients. The study has 80–90% power to detect a 25 relative risk reduction for the primary outcome of CPSP at 3 months at a significance level (alpha) of 0.05 (two-sided), anticipating a 50–60% CPSP rate in the control (inhalation or TIVA) arm with an allowance of 10% of patients lost to follow-up, or withdrawn or withdrawing from the study.

#### Statistical analyses

Data will be expressed as means ± standard deviation or numbers (percentages). Baseline characteristics will be compared using chi-squared or Fisher’s exact tests, a Student’s *t* test, or a nonparametric test as appropriate. The primary outcome, the occurrence of CPSP at 3 months, 6 months, and 1 year after surgery, will be compared using chi-squared or Fisher’s exact tests, and the relative risks and the 95% confidence interval will be calculated. All analyses of primary outcomes will be conducted using the intention-to-treat approach. A sensitivity analysis using the per-protocol approach will also be performed. In addition, a multiple logistic regression analysis will be used to identify relevant baseline covariates associated with the primary outcome. Variables tested in the model will be selected if *P* < 0.10 or if they are clinically relevant (such as usage of analgesics during surgery). All secondary outcomes are continuous variables and will be compared using the unequal-variance Student’s *t* test. Results are considered statistically significant if *P* < 0.05. Statistical analyses are performed using statistical software SPSS 17.0.

### Participant timeline

Recruitment of patients and data collection started in February 2019. Sufficient participants have been enrolled (500 patients) at the end of June 2019. The 1-year postoperative follow-up will be completed in June 2020.

### Data management and monitoring

All original data will be recorded in case report forms. The study supervisor (Hai Yu) will supervise the conduct of the trial conduction and perform monthly audits of the trial.

## Discussion

To our knowledge, this is the first prospective randomized controlled trial to investigate the prevention of CPSP following cardiac surgery through sternotomy for patients receiving volatile anesthesia compared to those receiving propofol-based TIVA using validated pain instruments in accordance with the IMMPACT recommendations.

CPSP is defined as persistent or recurrent pain lasting longer than 3 months and without an apparent cause [32]. Our results are clinically important because the incidence of CPSP can occur in 11–56% of patients undergoing cardiac or thoracic surgery with a median sternotomy, and currently there are established methods of prevention [10–12]. As we know, general anesthesia is routinely used in cardiac surgery. To date, it is well established that propofol can improve postoperative analgesia compared with inhalational anesthesia [16, 17]. As acute pain is viewed as an initial phase of the pain response that has the potential to progress to chronic pain [5], it was assumed that propofol has the potential to prevent CPSP, though it has conflicting roles. Ogurlu et al. reported that general anesthesia with propofol was associated with reduced persistent pain at 3 months compared to sevoflurane-based anesthesia, among patients undergoing open abdominal hysterectomy [18]. Moreover, Song et al. found consistent results in patients who had undergone a thoracotomy during surgery for lung and esophageal cancer [19].

However, there is no information in the literature on the relationship between the type of anesthesia used in cardiac surgery and subsequent chronic pain. To date, no clinical trial has been published comparing the effects of volatile anesthesia with those of propofol-based TIVA on postoperative acute and chronic pain after cardiac surgery through sternotomy.

On the other hand, prior studies focusing on CPSP after either cardiac or non-cardiac surgery assessed pain using a yes or no scoring system or the NRS evaluation of the intensity of pain. Few studies have used the IMMPACT recommendations [20]. IMMPACT is a consortium of researchers and practitioners in pain medicine whose mission is to develop consensus reviews and recommendations for improving the design, execution, and interpretation of clinical trials of treatments for pain [33]. The domains of pain recommended by IMMPACT show the diversified effect of anesthetics on pain at rest, pain during activity, the quality of pain, and the physical and emotional impact of pain.

In summary, this randomized controlled trial may provide important information on the influence of these two anesthetic regimens on CPSP after cardiac surgery. Moreover, using validated pain instruments to measure CPSP in accordance with the IMMPACT recommendations will add to the currently available data on CPSP, which is important since CPSP is clinically meaningful and disturbs patients’ physical and emotional functioning and quality of life.

## Supplementary information

**Additional file 1.** SPIRIT 2013 checklist.

**Additional file 2.** SPIRIT figure.

## Data Availability

Not applicable.

## Abbreviations

CPB: Cardiopulmonary bypass
CPSP: Chronic postsurgical pain
EuroSCORE: European System for Cardiac Operative Risk Evaluation
IMMPACT: Initiative on Methods, Measurement, and Pain Assessment in Clinical Trials
IV: Intravenous
NMDA: N- methyl-D-aspartate
NRS: Numerical rating scale
QoR-15: 15-item Quality of Recovery Questionnaire
SPIRIT: Standard Protocol Items: Recommendations for Interventional Trials
TIVA: Total intravenous anesthesia
