# Supplementary material for: Influence of volatile anesthesia versus total intravenous anesthesia on chronic postsurgical pain after cardiac surgery using the Initiative on Methods, Measurement, and Pain Assessment in Clinical Trials criteria: study protocol for a prospective randomized controlled trial
Source: Trials. 2019 Nov 27;20:645. doi: 10.1186/s13063-019-3742-4 (PMC6880356; doi:10.1186/s13063-019-3742-4)
Supplement: Supplementary file 2 — Additional file 2. SPIRIT figure. [file 13063_2019_3742_MOESM2_ESM.pdf]

	STUDY PERIOD								
	Enrollment	Allocation	Intervention	Post-intervention					
Time point	Preoperative visit	Allocation	During surgery	POD 1	POD 2	POD 3	3 months	6 months	1 years
ENROLLMENT:									
Eligibility screen	×								
Informed consent	×								
Demographic data	×								
Allocation		×							
INTERVENTIONS			×						
Volatile anesthesia			×						
TIVA			×						
Adverse events			×						
Surgery and anesthesia data			×						
ASSESSMENTS:				×	×	×	×	×	×
11-point NRS scale				×	×	×			
Opioid consumption				×	×	×			
IMMPACT criteria							×	×	×
Brief pain inventory							×	×	×
McGill questionnaire							×	×	×
QoR-15 questionnaire							×	×	×
